# The Overlap of Dietary Supplement and Pharmaceutical Use in the MIDUS National Study

**DOI:** 10.1155/2014/823853

**Published:** 2014-04-16

**Authors:** David S. Kiefer, Joe C. Chase, Gayle D. Love, Bruce P. Barrett

**Affiliations:** ^1^Department of Family Medicine, University of Wisconsin, 1100 Delaplaine Court, Madison, WI 53715, USA; ^2^Institute on Aging, University of Wisconsin, 1100 Delaplaine Court, Madison, WI 53715, USA

## Abstract

*Introduction*. In the United States, dietary supplement (DS) use is common, often takes place outside of the purview of health care providers, and may involve DS in combination with pharmaceuticals. This situation has led to concerns about interactions between DS and pharmaceuticals, as well as the risks from polypharmacy and polysupplement use. *Methods*. We used data from the Midlife in the US study (MIDUS 2 Survey) to examine DS and prescription pharmaceutical use in 3876 study participants in order to determine the demographics of high-users (5 or more) of DS and pharmaceuticals and the presence of DS-pharmaceutical co-use. *Results*. Over 69% of study participants regularly used DS, 49.6% regularly used both DS and pharmaceuticals, and 6.3% and 8.7% were high-users of pharmaceuticals and DS, respectively. High-users of DS, pharmaceuticals, and either were more likely than the whole cohort to be female and of lower income. *Conclusions*. These findings corroborate those of other national studies with respect to the demographics of DS users but add new information about people at risk of DS-pharmaceutical interactions, not an insignificant proportion of the population examined by this dataset.

## 1. Introduction

In the United States (USA), the use of complementary and alternative medicine (CAM) is common [1]. One component of CAM is a category referred to as dietary supplements (DS), which includes herbal medicines, vitamins, minerals, and other substances such as amino acids and enzymes [2].

Nationwide surveys, including Midlife in the United States (MIDUS), have begun the process of delineating the demographics of DS users, the prevalence of DS use, and other related factors such as disclosure to health care providers (HCP) and sources of DS information (Table 1). The results of such surveys show that the use of DS is not insignificant, with estimates of 20% of the US population regularly using DS [1, 3]. These rates may be even higher in some groups such as immigrant populations [4]. In recent years, the medical literature has also begun the process of assessing DS efficacy and safety, including issues surrounding the ingestion of numerous DS [5, 6], adverse dietary supplement-pharmaceutical interactions [3, 7–9], and specific DS-pharmaceutical combinations that warrant extra caution on the part of HCP [10].

With respect to the use of multiple DS, there is no generally accepted threshold at which extra risk is thought to occur, though there is some guidance about this topic in the polypharmacy literature. Polypharmacy is a situation of high risk for adverse interactions or drug effects, often defined as the simultaneous ingestion of five or more pharmaceuticals, though the most accurate determination of risk for a given individual would also take into effect other factors such as their medical history and the appropriateness of a pharmaceutical or pharmaceutical dose [11, 12]. The assessment of DS safety involves a careful examination of interactions between DS and pharmaceuticals to identify any adverse health outcomes associated with co-use [13, 14]. All of these concerns are heightened when health care providers are unaware of DS use, a common phenomenon in the USA, with data showing that nondisclosure rates approach 70% in some populations [7, 15–18].

From its inception, MIDUS, a longitudinal study of health and aging, has included a wide array of demographic and psychosocial measures as well as comprehensive assessments of health (physical and mental) and health behaviors including use of prescription pharmaceuticals (Rx) and over-the-counter medications (OTC) [19, 20]. In the first longitudinal follow-up (MIDUS 2, 2004–2006), assessments of medication use were expanded to include DS, thus creating a dataset containing detailed information about Rx, DS, and OTC use.

The aim of this analysis was to add to the DS literature by analyzing data from the MIDUS 2 survey in order to (1) improve knowledge surrounding the characteristics of users of DS, comparing these results to other large national surveys, and (2) explore the presence DS-Rx co-use and the demographics of people involved.

## 2. Participants and Methods

At baseline (MIDUS 1, 1995-1996) study participants (*n* = 7108) were noninstitutionalized, English-speaking adults in the continental USA, aged 25–74 years. As described elsewhere [20], the MIDUS 1 sample was comprised of 3 subsamples: the Main sample recruited using random-digit dialing methods (*n* = 4244), siblings of Main sample participants (*n* = 950), and a national sample of twins (*n* = 1914), all of whom were invited to complete telephone interviews and self-administered questionnaires. Nine to ten years after MIDUS 1 (in 2004-2005), these individuals were invited to participate in the MIDUS 2 survey, which included a phone interview and another self-administered questionnaire [20]. Medication use is assessed in the self-administered questionnaire, while the demographic data is obtained via the telephone interview. Only a subset of individuals (*n* = 4,006) who completed both the telephone survey and the self-administered questionnaire at MIDUS 2 were included in the current analysis; this subsample is not significantly different from the larger sample from which it is drawn [21]. Of the 4,006 study participants, only 3,876 completed study questions pertaining to DS and Rx use.

The demographic variables of interest are age in years, gender, educational level (less than high school or high school graduate (HS) or equivalent (GED), some college, and college graduate or more), and personal income, reported by the respondent as wages over the last year, an estimate of a person's financial resources and status.

The MIDUS 2 self-administered questionnaire included two sets of items assessing Rx and DS use: (1) “During the past 30 days have you taken prescription medicine for any of the following conditions?” Individuals were classified as pharmaceutical users if they said “yes” to at least one of these items; (2) “Please check below any of the following vitamin, mineral, or herbal supplements you take regularly—that is, at least a couple of times a week.” The DS checklist included common herbal medicines, vitamins, and minerals and provided study participants the option to add DS not found on the list. Individuals were classified as DS users if they checked at least one item on this list. In addition, the total numbers of DS and Rx being used were tabulated.

Four categories of DS and Rx users were created: (1) neither DS nor Rx used; (2) Rx only in the past 30 days; (3) DS only used regularly; and (4) both DS and Rx used. Furthermore, three categories of “high-users” were created: (1) using five or more Rx in the past 30 days (regardless of DS use); (2) using five or more DS regularly (regardless of Rx use); and (3) using five or more of* either* DS* or* pharmaceuticals. This distinction is designed to capture study participants who were in a polypharmacy and/or polysupplement situation.

## 3. Data Analysis

Descriptive statistics of individual and paired variables were examined in tabular and graphic format. A chi-squared analysis for proportion was utilized to compare variables as detailed in Tables 2 and 3. Any* z*-test* P* value <0.05 was considered statistically significant; values meeting these criteria were labeled as such in the relevant tables. Logistic regression analysis was then used to evaluate the group differences between high- and low-users of DS and Rx. All analyses used SPSS (IBM SPSS Version 21, 2012). Missing data was deleted from the cases of interest for the DS and pharmaceutical variable analyses.

## 4. Results

**Figure 1:**
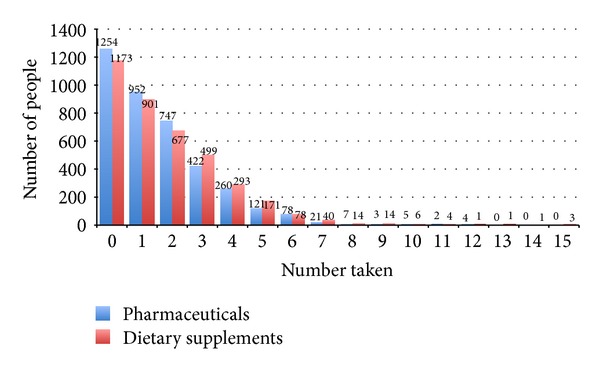
Number of people in MIDUS 2, Project 1, ingesting a given number of prescription pharmaceuticals (in the past 30 days) or dietary supplements (“regularly”).

Demographic characteristics for the full sample, as well as categories of DS and pharmaceutical use, are summarized in Table 2. The sample is predominantly female (55.7%), aged 56.2 years on average, with a mean income of approximately $28,000 and having a high school education or less. In addition, among the 3,876 study participants, 2,703 (69.7%) were taking at least one DS recently, 2,622 (67.6%) were taking at least one pharmaceutical in the last 30 days, 1,923 (49.6%) were taking both, and 474 (12.2%) were taking neither (Figure 1).

When compared to the entire cohort (*n* = 3876), there were statistically significant differences in the demographics of study participants using neither, any Rx, any DS, or both DS and Rx (Table 2, columns 3–6). Notably, when compared to the whole cohort, people using neither were less likely to be female (55.7% versus 43.3%, resp.) and were younger (mean age 56.2 versus 48.7), with a higher income (about $28,000 annually versus about $38,000). In contrast, users of both DS and Rx were more likely to be women (62.3%) and were older (mean age 60.2), with a lower income ($16,000–$17,999). Those study participants who used any DS (column 5) were slightly older than the whole cohort (mean age 58.0 versus 56.2, resp.), more likely to be female (59.4% versus 55.7%, resp.), and had a slightly higher income ($32,500–$34,999 versus $27,500–$29,999), though their education was similar.

When neither category was used as the comparison, both DS users and Rx users were older (average age 48.7 versus 58.0 and 58.6, resp.), while the Rx users had more people in the high school or less education category (31.0% versus 36.2%, resp.).

**Figure 2:**
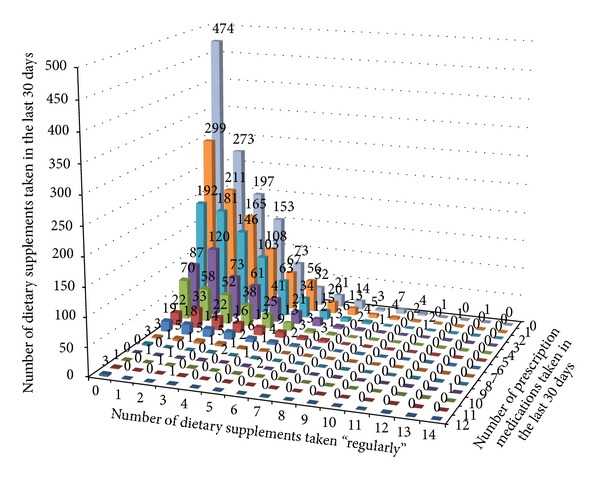
The MIDUS 2 Project 1 cohort (*n* = 3876): the number of people taking a given number of dietary supplements (0–15) and prescription medications (0–12).

The number of DS and Rx taken by study participants is shown in Figure 1. Study participants ingested between 0 and 15 DS and 0 and 12 Rx. As mentioned above, nearly half the sample (49.6%) were taking both DS and Rx; a cluster exists in the lower numbers of DS and Rx, though individuals populate even the higher number combinations (Figure 2). Examples of these higher number combinations are one person taking nine Rx and 14 DS, one person taking five Rx and nine DS, one person taking three Rx and 11 DS, and one person taking two Rx and 11 DS (Figure 2).

The demographics of high-users (≥5) of DS (*n* = 333), Rx (*n* = 241), or either DS or Rx (*n* = 546) are presented in Table 3. When compared with the total sample (*n* = 3876), people in all three categories were more likely to be female, older (mean age 65 or greater), and with a lower median income. The low annual income result for the high-user Rx group was affected by respondents answering “zero” to wages over the last year. With respect to education, DS users tend to have more education and Rx users less education. In the high-user Rx group, more people had high school or less education, and less people had a bachelor's degree or more education, when compared to the high-user DS group. Of note, 28 individuals who were high-users of* both* DS and Rx were identified, so ≥5 DS and ≥5 Rx (Table 3, column 6). Exploring the demographics of the high-users of either DS or Rx (Table 3, column 5), a logistic regression illustrated that female gender, lower income, and higher age make it more likely that a study participant is in the “high-use” category, whereas amount of education was less of a determinant.

## 5. Discussion

Large national datasets provide information that can answer questions of importance to health care delivery and decision-making. This is no exception with MIDUS 2 survey, which shows that people in this dataset use DS and pharmaceuticals simultaneously and in multiple quantities. The results presented here both corroborate past research and provide an expansion of the topic by exploring details behind DS and pharmaceutical co-use. For example, this analysis illustrates that recent DS users (any quantity) are more likely to be older and women, in line with other prior national surveys such as the 2007-2008 update to the National Health and Nutrition Examination Survey (NHANES) and the National Health Interview Survey (NHIS) [1, 3, 22, 23]. In contrast, this analysis showed a similar amount of education between the DS cohort and the whole cohort; in other trials, DS users are often more likely to be more highly educated. Of note, the MIDUS study participants are considered highly educated at baseline [20, 21], perhaps affecting any additional education effect that might appear in subanalyses.

As presented in Table 3, the data on users of five or more DS and/or Rx (“high-users”), considered to be an important high-risk group, shows that high-users tend to be older and female. Of the high-user groups, high-users of DS and both DS and Rx had more education, whereas high-users of pharmaceuticals had less education overall. Picking apart the meaning and etiology of these trends, and finding clinical relevance, is a challenge. Clearly, women, in particular, women at a higher average age, are at risk of adverse dietary supplement-pharmaceutical interactions because they are users of both DS and pharmaceuticals in high numbers; this is a demographic worthy of a clinician's attention in this respect.

With respect to the income variable, there are several reasons why it is more difficult to draw clinically relevant conclusions. For example, more than for other variables, in MIDUS 2 there is missing income information, affecting the statistical significance of the results. Also, with increasing age, income is replaced by retirement funds, not necessarily captured by the survey questions which focus on reportable wages; this would artificially convey that someone has a lower income when they may, in fact, have significant regular retirement income. Future analyses to examine other variables in the MIDUS 2 survey dataset relevant to income, such as retirement income, will help to further examine this variable and allow a closer comparison to the NHANES results that showed a higher income in DS users.

There are several additional study limitations that could have affected the results presented and their generalizability. For example, the MIDUS 2 survey has a small percentage of people of nonwhite races and ethnicities, restricting its generalizability to the US population. Furthermore, our analysis did not include iron nor calcium supplements, even those that have been included in some, but not all, other national surveys. These variables were separate from the DS data, though still part of the MIDUS 2 dataset, and there is debate about whether or not such minerals should be considered DS. If anything, the inclusion of calcium and iron in our analysis would have further increased the DS use data for women and older individuals, given that such products are not uncommonly used in that population. Along the same lines, the questions in this survey included multivitamins as part of DS, similar to some, but not all, prior surveys (Table 1). In MIDUS 2, it is not possible to separate out multivitamin use from other DS; multivitamin users may in fact represent a different demographic from other DS users, though it is not possible to comment on this using these results.

It was beyond the scope of this analysis to include the use of over-the-counter medications, nor the specific pairings of DS with diagnoses and health parameters. Some of this data is contained in MIDUS 2, but an expansion of this information exists in MIDUS 2 Biomarker data; future analyses intend to explore these aspects of the DS-pharmaceutical overlap. The specific DS being used and overlaps with pharmaceuticals for individual study participants is an important next step in focusing efforts in a targeted way on decreasing the most concerning adverse dietary supplement-pharmaceutical interactions. In addition, numerous other variables could be involved with whether people use DS, RX, or both. One example is insurance coverage; NHIS found that DS use was higher in people with no insurance. Given that the analysis presented here found differences between the cohort as a whole and users (“any” and “high”) of both DS and Rx, future analyses are intended to determine which other factors, such as insurance, are involved.

In summary, this analysis provides some insight into the demographics of DS users, pharmaceutical users, high-users or either, and those at risk of adverse DS-Rx due to co-use for the MIDUS 2 survey dataset. This large national survey shows that a not insignificant percentage of people are taking both DS and Rx and that there are important contributions to this group from gender status, age, education, and income. Merely at the beginning of the process of identifying who might be at risk for adverse dietary supplement-pharmaceutical interactions, this study illustrates a method that could be used in other large national surveys and datasets with DS and pharmaceutical data and serves as a reminder to clinicians to be aware of such co-use in some patients more than others, but ideally in all demographics. With that being said, the ideal way to prevent polypharmacy, polysupplement use, and adverse dietary supplement-pharmaceutical interactions would be to query every patient about DS use and have an informed discussion about risks and benefits in the context of their health cosmology, past medical history, and pharmaceutical use. In that way, each of the data points in Figure 2 would receive attention in the clinical setting.

## Figures and Tables

**Table 1:** Demographics and DS use in four national surveys.

	National Health and Nutrition Examination Survey (NHANES)	National Health Interview Survey (NHIS)	American Association of Retired Persons (AARP)	Midlife in the United States (MIDUS 2)
Years	2007-2008	2002, 2007	2006	2004–2007
Number of participants	3364	30,427 (2002), 22,657 (2007)	1559	5895
Ages	20–69	18+	50+	35–86
DS included*	HM, M, V, O	HM, O	HM, O	HM, M, V, O
% using DS	47.7	17–19	42	69.7%
Reference	Kennedy et al., 2013 [22]	Wu et al., 2011 [23]; Barnes et al., 2009 [1]; Hanyu et al., 2002 [24]	AARP and the National Center for Complementary and Alternative Medicine, 2007 [25]	Dienberg Love et al., 2010 [21]; Radler and Ryff, 2010 [20]

*DS: dietary supplement; HM: herbal medicine, M: minerals, V: vitamins, O: other dietary supplements.

**Table 2:** Demographic profiles for study cohort as a whole and with respect to use or nonuse of dietary supplements (DS) and pharmaceuticals (Rx).

Demographic characteristic	Total sample (*n* = 3876)	Neither DS nor Rx (*n* = 474)	Any Rx (*n* = 2622)	Any DS (*n* = 2703)	Both DS and Rx (*n* = 1923)
Gender					
Female (%)	55.7	43.3^a^	59.4^ab^	59.4^ab^	62.3^abc^
Mean age (SD)	56.2 (12.4)	48.7 (10.2)^ab^	58.6 (12.3)^ab^	58.0 (12.2)^ab^	60.2 (11.9)^abcd^
Median income	$27,500–$29,999	$22,000–$22,499	$22,000–$22,499	$32,500–$34,999	$16,000–$17,999
Education (%)					
HS-GED or less	33.2	31.0	36.2^a^	31.1	34.0^d^
Some college	28.8	27.6	29.1	28.2	29.9
College or more	38.0	41.4	34.7^ab^	39.1	36.1^bd^

^a^ *P* < 0.05 when compared to the total sample (column 2).

^b^ *P* < 0.05 when compared to “neither using DS (regularly) nor pharmaceuticals (in the last 30 days)” (column 3).

^c^ *P* < 0.05 when compared to “any Rx” (column 4).

^d^ *P* < 0.05 when compared to “any DS” (column 5).

**Table 3:** Demographic profiles (in %) for “high-users” (≥5) of prescription pharmaceuticals (Rx), “high-users” (≥5) of dietary supplements (DS), and study participants using ≥5 either Rx or DS.

Demographic characteristics	Total sample (*n* = 3876)	“High-users” of Rx in the past 30 days (*n* = 241)	“High-users” of DS “regularly” (*n* = 333)	“High-users” of Rx or DS (*n* = 546)	“High-users” of Rx and DS (*n* = 28)
Gender					
Female	55.7	68.5^a^	65.2^a^	66.7^a^	35.7%^a,b^
Mean age (SD)	56.2 (12.4)	61.7 (11.3)^a^	59.2 (11.3)^ab^	60.0 (11.4)^a^	65.0 (10.2)^a^
Median income	$27,500–$29,000	$2,000–$3,999	$18,000–$19,999	$10,000–$11,999	$1,000–$1,999
Education					
HS-GED or less	59.7	52.7^a^	25.8^ab^	37.0	39.3^a^
Some college	22.0	31.1	29.7	29.9	39.3^a^
Bachelors+	18.2	16.2^a^	44.4^ab^	33.2^a^	21.4

^a^ *P* < 0.05 when compared to the total sample (column 2).

^b^ *P* < 0.05 when “high-users” of DS are compared to “high-users” of prescription pharmaceuticals.
